# Scoping review of managed alcohol programs

**DOI:** 10.1186/s12954-022-00646-0

**Published:** 2022-07-25

**Authors:** Shannon M. Smith-Bernardin, Leslie W. Suen, Jill Barr-Walker, Isabel Arrellano Cuervo, Margaret A. Handley

**Affiliations:** 1 School of Nursing, Department of Social and Behavioral Sciences, University of California, San Francisco, San Francisco, CA USA; 2 UCSF Benioff Homelessness and Housing Initiative at ZSFG Hospital and Trauma Center, San Francisco, CA USA; 3 National Clinician Scholars Program, Philip R. Lee Institute of Health Policy Studies, University of California, San Francisco, San Francisco, CA USA; 4 San Francisco Veterans Affairs Medical Center, San Francisco, CA USA; 5 ZSFG Library, University of California, San Francisco, San Francisco, CA USA; 6 Contra Costa County Family Medicine Residency, Martinez, CA USA; 7 Department of Epidemiology and Biostatistics, University of California, San Francisco, CA USA; 8 PRISE Center: Partnerships for Research in Implementation Science for Equity, University of California, San Francisco, CA USA

**Keywords:** Managed alcohol program, Harm reduction, Alcohol use disorder, Homelessness, Scoping review

## Abstract

**Background:**

Internationally, strategies focusing on reducing alcohol-related harms in homeless populations with severe alcohol use disorder (AUD) continue to gain acceptance, especially when conventional modalities focused on alcohol abstinence have been unsuccessful. One such strategy is the managed alcohol program (MAP), an alcohol harm reduction program managing consumption by providing eligible individuals with regular doses of alcohol as a part of a structured program, and often providing resources such as housing and other social services. Evidence to the role of MAPs for individuals with AUD, including how MAPs are developed and implemented, is growing. Yet there has been limited collective review of literature findings.

**Methods:**

We conducted a scoping review to answer, “*What is being evaluated in studies of MAPs? What factors are associated with a successful MAP, from the perspective of client outcomes? What are the factors perceived as making them a good fit for clients and for communities?*” We first conducted a systematic search in PubMed, Embase, PsycINFO, CINAHL, Sociological Abstracts, Social Services Abstracts, and Google Scholar. Next, we searched the gray literature (through focused Google and Ecosia searches) and references of included articles to identify additional studies. We also contacted experts to ensure relevant studies were not missed. All articles were independently screened and extracted.

**Results:**

We included 32 studies with four categories of findings related to: (1) client outcomes resulting from MAP participation, (2) client experience within a MAP; (3) feasibility and fit considerations in MAP development within a community; and (4) recommendations for implementation and evaluation. There were 38 established MAPs found, of which 9 were featured in the literature. The majority were located in Canada; additional research works out of Australia, Poland, the USA, and the UK evaluate potential feasibility and fit of a MAP.

**Conclusions:**

The growing literature showcases several outcomes of interest, with increasing efforts aimed at systematic measures by which to determine the effectiveness and potential risks of MAP. Based on a harm reduction approach, MAPs offer a promising, targeted intervention for individuals with severe AUD and experiencing homelessness. Research designs that allow for longitudinal follow-up and evaluation of health- and housing-sensitive outcomes are recommended.

## Introduction

Alcohol-related deaths are on the rise in the USA, with 72,558 deaths reported in 2017, more than double the number from 1999 [1]. Worldwide, there were nearly 3 million deaths related to the harmful use of alcohol, with increases seen throughout the COVID-19 pandemic [2–5]. Additionally, over 30% of countries surveyed by the WHO have policies and efforts related to the consumption of “surrogate alcohol” (i.e., non-beverage alcohol (NBA) such as hand sanitizer, rubbing alcohol, and mouthwash) [2]. Approximately 10.6 million adults in the USA had alcohol use disorder (AUD) in 2017 [6]. Adults experiencing homelessness are especially at risk, with AUD prevalence estimates exceeding 50%, and alcohol and drug use contributing to one-third of deaths in adults experiencing homelessness in one study [7, 8]. Communities experiencing both homelessness and severe AUD (a condition defined by the presence of six or more symptoms of AUD as described in the Diagnostic and Statistical Manual V) [9] experience high rates of physical and emotional trauma, severe medical comorbidities and mental illness, and difficult social circumstances that can be barriers to achieving stable housing and long-term alcohol abstinence. Internationally, harm reduction strategies have gained traction by focusing on the reduction in alcohol-related harms in populations experiencing co-occurring homelessness and AUD, especially in those where conventional modalities focused on alcohol reduction and abstinence have not been successful. One such strategy is the managed alcohol program (MAP), a non-abstinence-based strategy managing the consumption of alcohol by providing eligible individuals with regular doses of alcohol as a part of a structured program, and often providing resources such as housing, access to medical care, regular meals, and other social services [10].

MAPs are founded on the principles of alcohol harm reduction. Compared to efforts aimed at reducing the harmful effects of drug use (e.g., syringe exchange, naloxone distribution) or HIV transmission (e.g., preventative HIV medication such as pre-exposure prophylaxis (PrEP)), alcohol harm reduction is less well known. Alcohol harm reduction has largely been approached at the population level [11, 12], balancing the challenge of modifying the consumption of a substance both legal and deeply ingrained in societal culture [2, 13–15]. And compared to abstinence-based efforts, alcohol harm reduction often includes policy efforts regarding access to and availability of alcohol at the population level and efforts to moderate consumption [11, 16–19]. At the same time, a subgroup of individuals with severe AUD likewise face structural oppression including economic disparities, poverty, homelessness, racism, and related stigmas [20–24]. The needs of this subgroup are typically not addressed, and may be negatively exacerbated, by reliance on population-level interventions to reduce moderate alcohol consumption [11]. MAPs operate at this intersection, addressing the co-occurring harms related to severe AUD and the vulnerability related to structural inequities and oppression.

At the time of this review, there were 38 established MAPs including programs located in both large and small project-based permanent housing, shelters, non-residential day programs, mobile services to scattered sites, COVID-19-related programs, and inpatient services [10, 25–28]. MAPs have existed since the late 1990s predominantly in Canada [10].

A primary goal of MAPs is to reduce the harmful effects related to alcohol consumption, paying particular attention to not increase or introduce additional harms. To reduce the harm from consumption of non-beverage products containing alcohol, MAPs provide safer sources of beverage alcohol to participants [10, 29, 30]. MAPs typically screen, provide care, and monitor physical and mental health conditions in collaboration with clients, increasing engagement and working to stabilize co-occurring conditions and reduce alcohol-related harms [10, 31].

To date, there has been no comprehensive review of MAP studies that evaluate the state of the research and collective findings internationally. To address this gap, we aimed to conduct a scoping review of both peer-reviewed and gray literature to answer the questions: *What is being evaluated in studies of MAPs? What factors are associated with a successful managed alcohol program, from the perspective of client outcomes?* And *what are the factors related to MAPs that are perceived as making them a good fit for clients and for communities?* We seek to inform researchers and public health officials on strategies and appropriateness for MAP development within health systems and communities. Our objectives were to identify the intended and measured impacts of a MAP including on client health outcomes, alcohol consumption, and housing stabilization, understand MAP feasibility and implementation, characterize the key components of MAPs, identify gaps in knowledge and the literature, and recommend possible areas for future study.

## Methods

### Search strategy

Our scoping review methodology followed Arksey and O’Malley [32] and Levac [33] frameworks and Preferred Reporting Items for Systematic Reviews and Meta-Analyses (PRISMA-ScR) guidelines [34, 35] (“Appendices 1 and 2”).

We used a three-step search process for identifying published and unpublished studies for our scoping review. First, a systematic search for articles involving programs that managed the consumption of alcohol was conducted in PubMed, Embase, PsycINFO, CINAHL, Sociological Abstracts, Social Services Abstracts, and Google Scholar on November 14, 2019, and updated on April 1, 2021. No date or language limits were used, and we included broad terms to identify a range of ways MAP programs may be characterized. We developed a search strategy in collaboration with a clinical librarian (JBW) using an iterative process that involved testing search terms, keywords, and controlled vocabulary, including MeSH and Emtree terms, and examining the relevance of corresponding search results. Detailed search strategies for each database can be found in “Appendix 3”. Next (Step 2), we searched the gray literature by developing focused Google and Ecosia searches on our topic. The gray literature search was conducted with the same search terms of the systematic search (noted in step one above) without date or language limitations. The first 100 web site results were opened and, if the result was not obvious, the site was investigated internally for the related search terms. Finally (Step 3), the reference lists of included articles were searched to identify additional studies, and the reviewers contacted experts to ensure that relevant studies were not missed.

### Study selection

Three reviewers (SSB, LWS, and IC) independently screened all articles from the original search results based on title and abstract and again for full-text review. All reviewers collaboratively reviewed screening decisions at each stage to ensure inter-rater reliability. One reviewer (SSB) independently screened the 64 articles from the search update. Studies were excluded if they did not contain full text of the article, were not in English, were not original research, were not focused on programs that managed or regulated the consumption of alcohol, focused on inpatient or emergency department hospitalization services only, included participants under 18, or did not include participants who were experiencing homelessness and AUD.

### Data extraction

A standardized form was created to extract data in the following areas: (1) study setting, (2) study type and methodology, (3) characteristics of the intervention and its implementation (e.g., intervention type, duration, and outcome measures used), and (4) findings and recommendations produced by the literature. In accordance with scoping review methodology, critical appraisal was not conducted [32, 33]. Data extraction was split by two reviewers (SSB and LWS) who each independently reviewed the extracted data for all included articles.

## Results

**Fig. 1:**
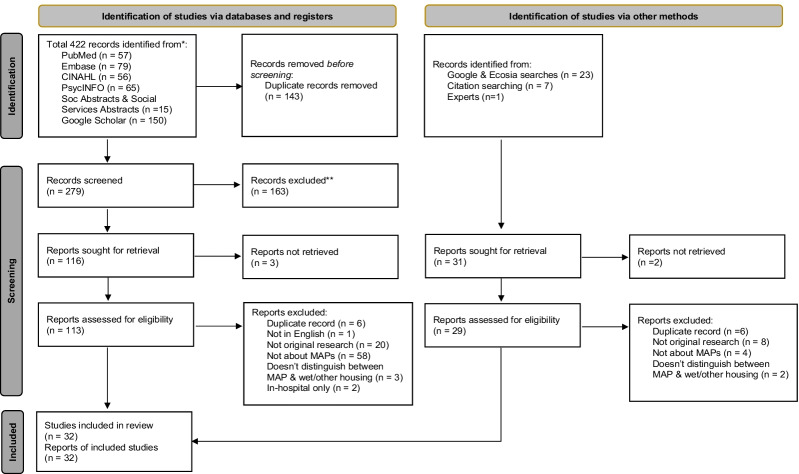
PRISMA flowchart of included studies in the review. *Consider, if feasible to do so, reporting the number of records identified from each database or register searched (rather than the total number across all databases/registers). **If automation tools were used, indicate how many records were excluded by a human and how many were excluded by automation tools. *From:* Page MJ, McKenzie JE, Bossuyt PM, Boutron I, Hoffmann TC, Mulrow CD, et al. The PRISMA 2020 statement: an updated guideline for reporting systematic reviews. BMJ 2021;372:n71. https://doi.org/10.1136/bmj.n71. For more information, visit: http://www.prisma-statement.org/

The literature search yielded 422 articles, and focused searching of gray literature, references, and communication with experts found an additional 31 articles. After excluding duplicates and screening 310 articles, 278 were eliminated because of their irrelevance to the topic. Thirty-two studies were included in the final review, as indicated in the PRISMA chart (Fig. 1) and Table 1.

**Table 1:** All articles included in scoping review

Author	Title	Year	Country	Methodological approach	Setting/MAP intervention	Sample size (MAP only)
*Primary data collection study designs*						
Podymow, T.; Turnbull, J.; Coyle, D.; Yetisir, E.; Wells, G	Shelter-based managed alcohol administration to chronically homeless people addicted to alcohol	2006	Canada	Pre–post evaluation within subject of shelter-based MAP program	Ottawa, Canada. MAP provides shelter with bed and meals, medication administration, daily RN and weekly MD visits, ADL support, care coordination	17 MAP program participants; 1 MAP program
Francoeur, N., Vachon, F., & Scott, G	No place like home: housing and harm reduction	2009	Canada	Cross-sectional interview, and survey on housing needs among attendees at one-day "Housing people experiencing persistent homelessness" workshop (*n* = 27 of 47 completed survey)	Waterloo, Canada	N/A
London Homeless Coalition	Managed alcohol: housing, health and hospital diversion—exploring a managed alcohol model for the city of London	2011	Canada	Cross-Sectional feasibility assessment for MAP based on interviews with persons with lived experience (*n* = 13), and consultations and semi-structured interviews with key informants (*n* = 43)	London, Ontario, Canada	N/A
Evans, J	Supportive measures, enabling restraint: governing homeless “street drinkers” in Hamilton, Canada	2012	Canada	Longitudinal cohort study with In-depth interviews, observation of program operations, and document review	Hamilton, Canada MAP based in residential housing with on-site physician and nursing care, counseling, social services, 24/7 support with activities of daily living	24 MAP participants; 2 MAP staff; 1 MAP program
Stockwell, T; Pauly, B; Chow, C; Vallance, K; Perkin, K	Evaluation of a managed alcohol program in Vancouver, BC: Early findings and reflections on alcohol harm reduction	2013	Canada	Pre–post evaluation of a pilot study with pre–post comparison within subject	Station Street MAP in Vancouver Canada implemented in 2011. MAP located in Housing first permanent housing/residence building	7 MAP participants; 1 MAP program
Evans, J.; Semogas, D.; Smalley, J. G.; Lohfeld, L	“This place has given me a reason to care”: Understanding “managed alcohol programs” as enabling places in Canada	2015	Canada	Qualitative study with interviews and focus groups	MAP in Ontario Canada that had been in operation for 5 years. 16 bed capacity for men. Provides permanent housing, shared rooms, three meals daily. Daily access to RN, registered practical nurses, and social service providers. Weekly access to family practice physicians	10 MAP participants; 1 program
Ezard, N; Dolan, K; Baldry, E; Burns, L; Day, C; Hodge, S; Cubitt, T; Loesch, B; Mackay, T	Feasibility of a managed alcohol program (MAP) for Sydney’s homeless	2015	Australia	Qualitative study with one-to-one structured interviews	Non-MAP: Interviews within 20-bed Gorman House in Sydney (residential unit with withdrawal management)	N/A
Hammond, K, Lynda G, Pauly, B	A cost–benefit analysis of a Canadian managed alcohol program	2016	Canada	Cost–benefit analysis with pre–post within-subject utilization comparison, and cost–benefit analysis with intervention (same as pre–post subjects) compared to control subjects	Kwae Kii Win Center in Thunder Bay, Ontario, Canada opened March 2012. 15-bed permanent supportive housing with communal living spaces. Residents have access to community worker (case manager), weekly NP visits, weekly Elder visits, community primary care, money mgmt, life skills training	18 MAP participants; 20 Control; 1 program
Pauly, B; Gray, E; Perkin, K; Chow, C; Vallance, K; Krysowaty, B; Stockwell, T	Finding safety: a pilot study of managed alcohol program participants’ perceptions of housing and quality of life	2016	Canada	Pre–post longitudinal evaluation with comparison within subject and comparison with treatment-as-usual controls. In-depth interviews with MAP participants and staff	Kwae Kii Win Center in Thunder Bay, Ontario, Canada opened March 2012. 15-bed permanent supportive housing with communal living spaces. Residents have access to community worker (case manager), weekly NP visits, weekly Elder visits, community primary care, money mgmt, life skills training	18 MAP participants; 20 Control; 1 program
Vallance, K.; Stockwell, T.; Pauly, B.; Chow, C.; Gray, E.; Krysowaty, B.; Perkin, K.; Zhao, J	Do managed alcohol programs change patterns of alcohol consumption and reduce related harm? A pilot study	2016	Canada	Pre–post longitudinal evaluation with comparison within subject and comparison with treatment-as-usual controls. In-depth interviews with MAP participants and staff	Kwae Kii Win Center in Thunder Bay, Ontario, Canada opened March 2012. 15-bed permanent supportive housing with communal living spaces. Residents have access to community worker (case manager), weekly NP visits, weekly Elder visits, community primary care, money mgmt, life skills training	18 MAP participants; 20 Control; 1 program
Grazioli, V., Collins, S., Paroz, S., Graap, C., Daeppen, J	Six-month outcomes among socially marginalized alcohol and drug users attending a drop-in center allowing alcohol consumption	2017	Switzerland	Cross-sectional design, no comparison group	Drop-in center in French-speaking area of Switzerland open 12-7 pm created related to lack of daytime shelters and public intoxication by socially marginalized adults; no housing or shelter provided. Capacity 25 clients. Staffing with social workers, nurses, and psychologist. Access to hygiene facilities (shower, laundry, toilets), clothing, nonalcohol beverages, snacks (free) and lunch (small fee). Counseling also provided during alcohol dosing. Attendance by participants is recorded to an administrative database	1 Day-Only MAP
Klingemann, J. & Klingemann, H	Drinking under control programs: perception of alcohol-related harm reduction measures in Poland. Results of a qualitative study among outpatient alcohol treatment providers	2017	Poland	Qualitative study with focus groups	N/A	N/A
Ramsperger, E. & Ramage, K	A selective literature review on managed alcohol programs and indigenous healing methodologies	2017	Canada	Evaluate feasibility and acceptability of MAP for Indigenous populations. Consultations/interviews. Conduct literature review of MAPs in Canada	Indigenous elders from Calgary and neighboring First Nation communities in Canada	N/A
Wettlaufer, A., Pauly, B., Brown, M., Chow, C., Vallance, K., Kauppi, C., Larocque, C., Stockwell, T., & Zhao, J	Toward alcohol harm reduction: results from an evaluation of a Canadian managed alcohol program	2017	Canada	Evaluation including monthly participant surveys x 6mos, and secondary data analysis, and in-depth interviews with select participants	Sudbury (Canada) Harm Reduction—Managed Alcohol Program. Day program only; no residential component	8 MAP participants; 16 Controls; 8 MAP staff; 1 MAP program
Beausoleil RM, Hunt-Jinnouchi F, Onespot-Whitney J, Brown M, Owens T	Indigenous pathways to health and well-being: managed alcohol program (MAP) feasibility study	2018	Canada	Survey assessing feasibility and acceptability of a MAP. No comparison group	N/A	N/A
Chow, C.; Wettlaufer, A.; Zhao, J.; Stockwell, T.; Pauly, B. B.; Vallance, K	Counting the cold ones: a comparison of methods measuring total alcohol consumption of managed alcohol program participants	2018	Canada	Comparison sample cross-sectional design	6 MAP programs: 2 in Ottawa, 1 each in Toronto, Hamilton, Thunder Bay and Vancouver. All have connection to housing with residential, transitional, or shelter-based models. Toronto: a 55-capacity shelter-based program for men upstairs from shelter for 543 homeless men. Ottawa: Features two programs for men and women: The Downtown MAP (12 beds) accepts new clients and assesses clients for a possible long-term stay at The Oaks facility (45 beds). Hamilton: A residential program for up to 22 men and women. Thunder Bay: A 15-bed residential facility for men and women. Vancouver: A 8-bed residential program for men and women	65 MAP participants; 6 MAP programs N/A
Ezard, N.; Cecilio, M. E.; Clifford, B.; Baldry, E.; Burns, L.; Day, C. A.; Shanahan, M.; Dolan, K	A managed alcohol program in Sydney, Australia: Acceptability, cost-savings and non-beverage alcohol use	2018	Australia	Qualitative study with one-to-one structured interviews	N/A	
Stockwell, T.; Pauly, B. B.; Chow, C.; Erickson, R. A.; Krysowaty, B.; Roemer, A.; Vallance, K.; Wettlaufer, A.; Zhao, J	Does managing the consumption of people with severe alcohol dependence reduce harm? A comparison of participants in six Canadian managed alcohol programs with locally recruited controls	2018	Canada	Comparison sample, cross-sectional survey design using survey data from participants	6 MAP programs: 2 in Ottawa, 1 each in Toronto, Hamilton, Thunder Bay and Vancouver. All have connection to housing with residential, transitional, or shelter-based models. Toronto: a 55-capacity shelter-based program for men upstairs from shelter for 543 homeless men. Ottawa: Features two programs for men and women: The Downtown MAP (12 beds) accepts new clients and assesses clients for a possible long-term stay at The Oaks facility (45 beds). Hamilton: A residential program for up to 22 men and women. Thunder Bay: A 15-bed residential facility for men and women. Vancouver: A 8-bed residential program for men and women	175 MAP participants; 189 Control; 6 MAP programs 5 MAP programs
Hall, S	Clients perspectives of managed alcohol programs in the first 6 months and their relational shifts	2019	Canada	Secondary data analysis of qualitative interviews utilizing interpretive description informed by constructivism	Five MAPs with connection to housing with residential, transitional, or shelter-based models. Toronto, Seaton House: men's-only shelter-based program with up to 114 men enrolled in MAP out of 543 shelter-capacity. Ottawa: Features two programs for men and women: The Wet Program MAP (12 beds) accepts new clients and assesses clients for a possible long-term stay at The Oaks facility (45 beds). Hamilton: A residential program for up to 20–24 men and women. Thunder Bay, Kwae Kii Win: A 15-bed residential facility for men and women	
Hedland, K. (Thesis)	Community programming in mental healthcare planning: a case study at the drinkers lounge in Vancouver, BC (Thesis)	2019	Canada	Qualitative interviews with narrative research methodology informed by methods of research of Indigenous populations	The Drinker’s Lounge is a small drop-in center in the Downtown Eastside (DTES) of Vancouver, run under the non-profit harm reduction organization, PHS Community Services Society (PHS). Focus on community building. Open M-F, 10a-2p. Tuesdays host weekly ‘meeting of the drinkers’. To become member, person must attend three Tuesday meetings	16 MAP participants; 2 MAP staff; 1 MAP program
Mattison CA, Belesiotis P, Wilson MG	Rapid synthesis determining the features of managed alcohol programs	2019	Canada	Literature review	Rapid synthesis of MAPs in Canada, including jurisdictional scan with findings detailing MAP staffing, operations, and funding	Basic details on 23 MAPs in Canada
Pauly, B; Brown, M.; Evans, J.; Gray, E.; Schiff, R.; Ivsins, A.; Krysowaty, B.; Vallance, K.; Stockwell, T	“There is a Place”: impacts of managed alcohol programs for people experiencing severe alcohol dependence and homelessness	2019	Canada	Multiple case study design utilizing situational analysis for 45–90-min semi-structured, in-depth interviews	6 MAP programs: 2 in Ottawa, 1 each in Toronto, Hamilton, Thunder Bay and Vancouver. All have connection to housing with residential, transitional, or shelter-based models. Toronto: a 55-capacity shelter-based program for men upstairs from shelter for 543 homeless men. Ottawa: Features two programs for men and women: The Downtown MAP (12 beds) accepts new clients and assesses clients for a possible long-term stay at The Oaks facility (45 beds). Hamilton: A residential program for up to 22 men and women. Thunder Bay: A 15-bed residential facility for men and women. Vancouver: A 8-bed residential program for men and women	57 current and former MAP participants; 50 MAP staff; 5 MAP programs N/A
Parkes, T.; Carver, H.; Matheson C.; Pauly, B.; Browne, T	Scoping the feasibility and acceptability of managed alcohol programs for people who are homeless with severe alcohol problems in community-based, third sector services in Scotland. Research briefing	2020	UK (Scotland)	Quantitative: case record reviews of those accessing third sector (civil society/not for profit) homelessness services who would meet the criteria for MAPs. Qualitative: semi-structured interviews (purposive sampling)	N/A	
Pauly, B; King, V.; Smith, A.; Tranquilli-Doherty, S.; Wishart, M.; Vallance, K.; Stockwell, T.; Sutherland, C	Breaking the cycle of survival drinking: insights from a non-residential, peer-initiated and peer-run managed alcohol program	2020	Canada	Semi-structured in-depth interviews with SEMAP participants recruited through purposive sampling	Vancouver-based Street Entrenched Managed Alcohol Program was operating as a non-residential community MAP and was entirely peer-developed and peer-led. The harm reduction goals for the MAP as identified by the peers were to help prevent withdrawal symptoms, improve daily functioning, and consume sufficient levels of alcohol to preclude the need for NBA. Specific goals included reduction in NBA use, and reduction in alcohol-related deaths	14 MAP participants; 1 MAP program
Carver, H., Parkes, T., Browne, T., Matheson, C., & Pauly, B	Investigating the need for alcohol harm reduction and managed alcohol programs for people experiencing homelessness and alcohol use disorders in Scotland	2021	UK (Scotland)	Quantitative: case record reviews of those accessing third sector (civil society/not for profit) homelessness services who would meet the criteria for MAPs. Qualitative: semi-structured interviews (purposive sampling)	N/A	N/A
T. Stockwell, T., Zhao, J., Pauly, B., Chow, C., Vallance, K., Wettlaufer, C., Saunders, J.B., & Chick, J	Trajectories of alcohol use and related harms for managed alcohol program participants over 12 months compared with local controls: a quasi-experimental study	2021	Canada	Quasi-experimental study within systematic longitudinal study with in-depth interviews of MAP participants (at 0–2, 6 and 12 months from admission) and of locally recruited controls	The six MAP programs included one site each in Toronto, Ottawa, Hamilton, Thunder Bay, Sudbury, and Vancouver. More detailed descriptions are provided in Pauly et al. (2018)	59 MAP participants;116 Controls; 6 MAP programs
*Secondary data studies*						
Kidd, S. A.; Kirkpatrick, H.; George, L	Getting to know Mark, a homeless alcohol dependent artist, as he finds his way out of the river	2011	Canada	Longitudinal single participant examination with interviews and chart review	20 bed MAP in Canadian urban center. Provides shelter, meals, treatment of medical and psychiatric conditions	1 MAP program participant
Pauly, B; Vallance, K; Wettlaufer, A; Chow, C; Brown, R; Evans, J; Gray, E; Krysowaty, B; Ivsins, A; Schiff, R; Stockwell, T	Community managed alcohol programs in Canada: overview of key dimensions and implementation	2018	Canada	Case study methodology with interviews, inductive content analysis	Study involves 13 community-based MAPs in 7 cities throughout Canada. The sites are not specifically named in the study. Programs included if: 1. An aim of program was to reduce harms; 2. There was daily alcohol dispensing for clients; and, 3. Alcohol was provided as part of the program. Excluded if: located in long-term care or hospital; tolerated alcohol use without any management of consumption, such as wet shelter	13 MAP programs
Erickson, R. A.; Stockwell, T.; Pauly, B. B.; Chow, C.; Roemer, A.; Zhao, J.; Vallance, K.; Wettlaufer, A	How do people with homelessness and alcohol dependence cope when alcohol is unaffordable? A comparison of residents of Canadian managed alcohol programs and locally recruited controls	2018	Canada	Cross-sectional comparisons of data on self-reported coping strategies collected from interviews	6 MAP programs: 2 in Ottawa, 1 each in Toronto, Hamilton, Thunder Bay and Vancouver. All have connection to housing with residential, transitional, or shelter-based models. Toronto: a 55-capacity shelter-based program for men upstairs from shelter for 543 homeless men. Ottawa: Features two programs for men and women: The Downtown MAP (12 beds) accepts new clients and assesses clients for a possible long-term stay at The Oaks facility (45 beds). Hamilton: A residential program for up to 22 men and women. Thunder Bay: A 15-bed residential facility for men and women. Vancouver: A 8-bed residential program for men and women	175 MAP participants; 189 Control; 6 MAP programs
Ristau, J.,Mehtani, N., Gomez, S., Nance, M., Keller, D., Surlyn, C., Eveland, J. & Smith-Bernardin, S	Successful implementation of managed alcohol programs in the San Francisco Bay Area during the COVID-19 crisis	2021	USA	Descriptive	San Francisco and Alameda Counties, California. I&Q: total 3 sites initially, to 7 total. Bed capacity 60–120 per site. Short-term up to ~ 16 days. Respite: 1 site. 75beds. I&Q staffing by disaster services workers: medical providers (MDs, NPs, PharmDs), public health nurses, behavioral health clinicians, and nonclinical support staff (including healthcare workers, clerical support workers, and shelter staff). At SF Respite SIP setting, staffing remained the same prior to COVID-19 and the implementation of the MAP pilot	21 MAP participants; 3 MAP sites
*Editorial/commentary*						
Muckle, J.; Muckle, W.; Turnbull, J	COMMENTARY: Operating principles from Ottawa’s managed alcohol program	2018	Canada	Descriptive	Downtown MAP & The Oaks (Ottawa Canada) operating since 2001. The 28 bed Downtown MAP transitional program operates as a starter site, focusing on stabilizing alcohol consumption, behavioral management, addressing care needs, and providing service linkages. The Oaks 55-bed program offers permanent housing, emphasizing life skill development, social connections, and is viewed as a graduate program of Downtown MAP for stabilized residents. Staffing includes client care workers, peer workers, nurses, physicians, and peer leaders (specifically at The Oaks)	1 MAP program
Stockwell, T. & Pauly, B	Managed alcohol programs: Is it time for a more radical approach to reduce harms for people experiencing homelessness and alcohol use disorders?	2018	Canada	Editorial	Editorial introduction to collection of studies of managed alcohol programs. Authors provide both benefits and risks of MAP related to alcohol harms, and insight to common concerns raised by reviewers and commentators responding to MAP research	N/A

Research on MAPs has progressed substantially in recent years, with most larger studies having been published in the last 5 years [10, 29, 30, 36–39]. The growing literature showcases a number of measured variables and outcomes of interest, with increasing efforts aimed at determining the effectiveness and potential risks of MAP.

Through our scoping review, we discerned four categories of findings in the literature that relate to: (1) the measurable client outcomes resulting from MAP participation (i.e., specific and measurable outcomes for health and harm reduction, utilization, and alcohol-related harms), (2) qualitative views and experiences from within a MAP; (3) feasibility considerations and fit of MAP development within a community; and (4) recommendations for implementation and evaluation, including collective lessons learned in the design, implementation, and evaluation of a MAP as detailed in the literature.

### Programs featured in the literature

There were 38 established MAPs found in this scoping review, including programs located in housing or shelters, non-residential day programs, mobile services to scattered sites, and inpatient services, of which 9 were featured in the research literature (Table 1). The majority of MAPs in operation are located within Canada; additional research works out of Australia, Poland, the USA, and the UK evaluate potential feasibility and fit of a MAP [40–44]. One study featured an additional three temporary MAP sites emerged during the COVID-19 pandemic response, including in short-term Isolation and Quarantine sites (intended for those with or exposed directly to COVID-19 to aid recovery and decrease community transmission) and within residential homeless programs such as medical respite/recuperative care [27]. An up-to-date directory of MAPs internationally can be found at https://www.uvic.ca/research/centres/cisur/assets/docs/resource-overview-of-MAP-sites-in-Canada.pdf.

### Measured outcomes

Our review found fourteen studies that evaluated quantitative MAP outcomes, including health and harm reduction outcomes, quality of life, alcohol consumption, housing retention, and utilization of services [27, 29–31, 37–39, 45–50]. Table 2 features quantitative findings.

**Table 2:** Quantitative outcomes on alcohol consumption, health outcomes, housing retention, and service utilization

Author, Year	Title	Location or country	Type of intervention	Sample size	Alcohol consumption	Health outcomes (acute and chronic)	Housing retention	Utilization of services	Other outcomes
Podymow, T.; Turnbull, J.; Coyle, D.; Yetisir, E.; Wells, G. (2006)	Shelter-based managed alcohol administration to chronically homeless people addicted to alcohol	Canada	Pre–post within subject	17 participants; 1 program	+	⟷	+	+	+Hygiene, nutrition + medication compliance
Stockwell, T; Pauly, B; Chow, C; Vallance, K; Perkin, K (2013)	Evaluation of a managed alcohol program in Vancouver, BC Early findings and reflections on alcohol harm reduction	Canada	Mixed-methods pilot study with pre–post comparison within subject	7 participants; 1 program	⟷ Beverage alcohol + NBA	⟷ Alcohol-related harms − Self-rated physical health − LFTs	+	⟷	+ Feasibility/acceptability
Evans, J.; Semogas, D.; Smalley, J. G.; Lohfeld, L. (2015)	“This place has given me a reason to care”: Understanding “managed alcohol programs” as enabling places in Canada	Canada	Qualitative study with interviews and focus groups	10 participants; 1 program	+	NR	+	NR	+ Interpersonal connections + Attention to physical health + Sense of self management, control of alcohol consumption
Hammond, K, Lynda G, Pauly, B (2016)	A cost–benefit analysis of a Canadian managed alcohol program	Canada	Cost–benefit analysis with pre–post within-subject utilization comparison, and cost–benefit analysis with intervention (same as pre–post subjects) compared to control subjects	18 MAP participants; 20 Control; 1 program	NR	NR	NR	+	+ Cost–benefit
Pauly, B; Gray, E; Perkin, K; Chow, C; Vallance, K; Krysowaty, B; Stockwell, T (2016)	Finding safety: a pilot study of managed alcohol program participants’ perceptions of housing and quality of life	Canada	Mixed-methods longitudinal pilot study with pre–post comparison within subject, and comparison with treatment-as-usual controls. In-depth interviews with MAP participants and staff	18 MAP participants; 20 Control; 1 program. Note 7 interviewees total for qualitative interviews	NR	NR	+	NR	+ Feasibility/Acceptability
Vallance, K.; Stockwell, T.; Pauly, B.; Chow, C.; Gray, E.; Krysowaty, B.; Perkin, K.; Zhao, J. (2016)	Do managed alcohol programs change patterns of alcohol consumption and reduce related harm? A pilot study	Canada	Mixed-methods longitudinal pilot study with pre–post comparison within subject, and comparison with treatment-as-usual controls. In-depth interviews with MAP participants and staff	18 MAP participants; 20 Control; 1 program	⟷ Beverage alcohol + NBA	+	NR	+	
Erickson, R. A.; Stockwell, T.; Pauly, B. B.; Chow, C.; Roemer, A.; Zhao, J.; Vallance, K.; Wettlaufer, A. (2018)	How do people with homelessness and alcohol dependence cope when alcohol is unaffordable? A comparison of residents of Canadian managed alcohol programs and locally recruited controls	Canada	Cross-sectional comparisons of data on self-reported coping strategies collected from interviews	175 MAP participants; 189 Control; 6 MAP programs	⟷ Beverage alcohol + NBA	NR	NR	NR	+ Coping strategies when alcohol is unaffordable
Stockwell, T.; Pauly, B. B.; Chow, C.; Erickson, R. A.; Krysowaty, B.; Roemer, A.; Vallance, K.; Wettlaufer, A.; Zhao, J. (2018)	Does managing the consumption of people with severe alcohol dependence reduce harm? A comparison of participants in six Canadian managed alcohol programs with locally recruited controls	Canada	Comparison sample, cross-sectional survey design using survey data from participants of 6 residential MAPs in 5 cities	175 MAP participants; 189 Control; 6 MAP programs	⟷ Beverage alcohol + NBA **only longer-term MAPs	+ Acute	NR	NR	
Evans, J. (2012)	Supportive measures, enabling restraint: governing homeless “street drinkers” in Hamilton, Canada	Canada	In-depth interviews, observation of program operations, and document review	24 MAP participants; 2 MAP staff; 1 MAP program	NR	+	+	NR	+ Relationship to alcohol
Wettlaufer, A., Pauly, B., Brown, M., Chow, C., Vallance, K., Kauppi, C., Larocque, C., Stockwell, T., & Zhao, J. (2017)	Toward alcohol harm reduction: results from an evaluation of a Canadian managed alcohol program	Canada	Small-scale mixed-methods evaluation	One MAP site. 8 MAP participants; 16 Controls; 8 MAP staff	+ Beverage alcohol − NBA	–	NR	NR	− Safety (within MAP) + Well-being
Stockwell, T., Zhao, J. Pauly, B., Chow, C., Vallance, K. Wettlaufer, A., Saunders, J.B., and Chick, J. (2021)	Trajectories of Alcohol Use and Related Harms for Managed Alcohol Program Participants over 12 months compared with local controls: a quasi-experimental study	Canada	Mixed-methods longitudinal pilot study with pre–post comparison within subject, and comparison with treatment-as-usual controls	MAP participants *n* = 59; Controls *n* = 116	+ Beverage alcohol + NBA	⟷ Alcohol-related harms ⟷ LFTs	NR	NR	More stringent rules for outside drinking = + outcomes for MAP participants Less stringent rules for outside drinking = MAP participants similar to controls for consumption, harms
Pauly, B; King, V.; Smith, A.; Tranquilli-Doherty, S.; Wishart, M.; Vallance, K.; Stockwell, T.; Sutherland, C. (2020)	Breaking the cycle of survival drinking: insights from a non-residential, peer-initiated and peer-run managed alcohol program	Canada	Qualitative–semi-structured in-depth interviews with SEMAP participants recruited through purposive sampling	*n* = 14 (all eligible persons identified participated)	+ NBA	+ Alcohol-related harms	NR	NR	

+ Significantly greater/improved in the intervention group (or post-intervention) = in favor of the intervention

↔No significant difference in pre- and post-intervention

−No statistical significance reported

?Significantly poorer in the intervention group (or post-intervention) = not in favor of the intervention

NR outcome not reported

There were ten studies evaluating health and/or harm reduction outcomes. Overall, study results suggested an improvement in quality of life among MAP participants who were less likely to report acute alcohol-related harms such as seizures, acute intoxication, trauma, or assault [29–31, 38, 39, 45, 47, 48, 51]. Notably, evaluation of alcohol-related harms found significantly fewer harms for participants of a Canadian MAP with more stringent policies on outside drinking (Stringent) as compared to controls (2.41 vs. 3.55, *p* < 0.01). Yet, participants in programs with more Lax policies (no rules regarding outside drinking or policies that were lax on outside drinking) had similar alcohol-related harm scores (3.22 vs. 3.55 *p* = 0.50) as controls [30]. Quality-of-life measures in addition to those alcohol-related were likewise assessed. In a single-site evaluation, MAP participants self-reported improvements in sleep, nutrition, health, and hygiene; via Diener's Satisfaction with Life Scale, participants scored a median of 22 equivalent to “slightly satisfied with life” [48]. Researchers assessed MAP participants as compared to treatment-as-usual individuals via the World Health Organization BREF scale (featuring 26 items covering four domains: physical, psychological, relationships, and environments) [38]. Compared to controls, MAP participants scored higher than controls (indicating higher quality of life) in all 4 domains; however, only the environmental domain was significantly higher. Within the environmental domain, MAP participants scored significantly higher than controls in five of seven scores: length of stay, safety, spaciousness, privacy, and overall quality [50]. Lastly, researchers evaluated the coping mechanisms utilized by three groups (newer MAP participants < 2 months, MAP participants for > 2 months, and shelter-based controls) when alcohol was not affordable [51]. They found longer-term (> 2 month) MAP participants had fewer negative coping behaviors (e.g., re-budgeting, theft from liquor store, other property theft, and consuming illicit drugs) than both newer MAP participants < 2 months and controls. Similarly, when faced with the unaffordability of alcohol, longer-term (> 2 month) MAP participants were more likely than newer < 2mos MAP participants and controls to utilize positive coping skills of seeking help or treatment. Longer-term MAP participants were also less likely to cope by going without alcohol [51].

There are a few ways to evaluate the biological impact of MAP, including measurement of liver function tests (LFTs). LFTs are measures within the blood that may indicate existing or worsening liver disease, a condition which may result from alcohol consumption. Improvement or stabilization in LFTs may provide substantial benefit to health by preventing often-fatal liver conditions including cirrhosis or acute liver failure. Three single-site evaluations from Canada included limited review of LFTs with pre–post within-participant evaluation. Two of these evaluations included participants with repeated LFTs (*n* = 5, and *n* = unknown as authors did not detail number of participants) indicated most showed persistent or worsening liver damage [45, 47]. Yet, authors of a third study (*n* = 13) found that of those with repeat measures all (*n* = 7/7) had persistent normal range or reductions in aspartate transaminase (AST), most (*n* = 9/10) had persistently normal or reduced alanine transaminase (ALT), and a single participant had a reduction in gamma-glutamyl transferase (GGT) [38]. A multi-site longitudinal evaluation showed promise that MAP participation itself does not negatively impact LFTs, yet departing a MAP may put the participant at higher risk for worsening LFTs [30]. The authors found albumin, a non-specific protein marker of liver function, decreased significantly during MAP participation, though to a small degree and well within normal ranges. Interestingly, they found a significant increase in AST when participants departed from MAP, both compared with their time within a MAP and before they entered. Bilirubin levels also increased in those participants who departed from the MAP. This may indicate MAPs should be long term to be protective and the positive benefits may end upon departure.

The authors of eight studies found that MAP participants reported a significantly reduced overall alcohol consumption compared to controls [29–31, 37–39, 46, 48]. This was possibly due to reduced NBA use, as two studies highlighted consistently reduced NBA consumption and NBA-using days in long-term MAP participants [29, 30, 38, 39, 45]. Authors assessed qualitative data and found MAP participants reported the predictable availability of alcohol allowed them to quell urges to binge drink and allowed transitions to stabilized drinking and/or periods of abstinence [29, 49]. MAP participants had greater number of drinking days, with one study finding an average of 27.8 alcohol days per month for MAP participants compared to 22.6 alcohol days for controls. Yet, this drinking pattern resulted both in a reduction in overall quantity of alcohol and in a less hazardous consumption across two Canadian studies [37, 38]. In general, the authors of one Canadian study found that it took about 2 months of MAP participation before drinking patterns stabilized [39].

However, several researchers have also cited concerns of participants under-reporting to staff the amount of alcohol consumed outside of MAP and possible underestimates of alcohol consumption in some programs, especially among those with lax policies around outside drinking [37, 38, 47]. Chow et al. from Canada found an average of 7.7 (range 2.7–9.9) outside alcoholic drinks consumed daily, on par with the level of alcohol being consumed within the MAP. Importantly, they found that significantly more standard drinks were reported to research personnel than MAP staff. This indicated that actual consumption patterns may be significantly different than those recorded within a MAP [37]. Addressing this disparity, Stockwell et al. evaluated non-MAP alcohol consumption for select Canadian participants at six MAP locations based on whether the MAP had more stringent outside drinking policies (“Stringent”) versus Lax [30]. Their findings indicate that, collectively, Canadian participants in both MAPs with Stringent and Lax policies had a higher number of drinking days in the last 30 days as compared to controls (patients not participating in any MAP). When distinguishing by outside drinking policies, participants in a Stringent model did have fewer alcoholic drinks per drinking day than controls (11.53 vs. 14.95, *p* = 0.3541, nonsignificant) and fewer NBA drinks per day (1.13 vs. 1.72, *p* = 0.04). However, those in a model with Lax outside drinking policies had significantly more drinks per day than controls (18.67 vs. 14.95, *p* = 0.02) and similar NBA drinks (2.17 vs. 1.72, *p* = 0.43) [39, 51].

Evidence for non-residential, day-only MAPs was limited to two Canadian programs, and studies were mixed regarding consumption outcomes [29, 46, 47]. Qualitative review of 1 day-only MAP program (open 4 h on weekdays) found that participants indicated a reduction in NBA on the days participating, yet an increase in NBA use and associated harms on weekends when the day program was unavailable [46]. In a second study, all participants of the peer-run day program who had consumed NBA prior to MAP participation stated reduction or complete cessation of NBA use [29]. This reduction was attributed to (1) secure and affordable alcohol supply and (2) a NBA trade-in program where the individual can receive beverage alcohol in trade for illicit alcohol-containing products [29]. Participants experiencing homelessness did state continued difficulty with both managing alcohol in the morning and evening hours outside of the MAP operations and, for those more remote from the day-program, difficulty in successfully traveling to the MAP [29, 46]. Considering quality of life, researchers out of Switzerland evaluated a drop-in, day-only option for alcohol consumption. They found that mental health quality-of-life scores measured via the French version of 12-Item Short Form Survey Instrument were significantly improved for participants with greater drop-in center attendance as compared to lower drop-in attendance [31].

Researchers of four studies examined housing retention, a finding that almost all participants were retained within the Canadian MAP after at least 5 months [45, 48–50]. In three single-site studies, MAP participants had 100% retention in housing during the study periods (*n* = 17, average retention 16 months; *n* = 10, average retention 42 months; *n* = 7, no average noted) [45, 48, 49]. Pauly et al. conducted a fourth single-site study and compared MAP participants to controls in an emergency shelter; *n* = 13/18 MAP participants retained housing throughout the 1-year evaluation period, while *n* = 20/20 controls remained homeless [50]. Ristau et al. conducted a descriptive evaluation of short-term COVID-19 Isolation and Quarantine sites in the USA (intended for a 10–14-day length of stay) and found that most MAP participants (*n* = 16/21) successfully completed quarantine with an average length of stay of 14.9 days [27].

Researchers of several studies also looked at service utilization of Canadian MAP participants and program cost analyses. They found mild-to-moderate reductions in emergency department visits, inpatient hospitalizations, and contacts with law enforcement [38, 48, 52]. Two cost analyses of Canadian MAPs found savings from reductions in acute care utilization and police encounters [48, 52]. Hammond et al. conducted a single study evaluating both within-subject changes and in comparison with treatment-as-usual controls in the emergency shelter system and found that savings from MAP participation outweighed program costs in Canada, resulting in savings of $1.21 per Canadian dollar invested per MAP participant compared to controls and $1.09 per Canadian dollar invested compared to pre-MAP within-subject utilization [52].

### Views from within a MAP

Our review found eleven studies representing 12 distinct MAPs (including a day-only program) qualitatively evaluating the individual experience within a MAP, including feasibility and acceptability, reconciling individual sense of self, community building, and changes in the client’s relationship to alcohol [36, 46, 49–51, 53–55].

In addition to measures of consumption, many participants noted a positive change in their relationship with alcohol, from a decreased focus on alcohol procurement to an increased feeling of control regarding consumption levels [49, 53–55]. Importantly, participants and staff noted the decrease in focus on alcohol led to increased sense of self-determination and motivation for positive change [36, 49, 50, 53, 55]. One study did note, as distribution of alcohol was essentially controlled by staff, participants expressed fear they would be unable to self-regulate alcohol consumption outside of a MAP [53]. A notable gap in the literature was the limited discussion related to other substance use, for both the effect of a MAP on previous drug use or ongoing drug use within a MAP in addition to alcohol consumption. In a needs assessment for a MAP in Scotland, findings indicated high levels of polysubstance use among the population identified as potentially appropriate for a MAP [42, 43]. Promising outcomes were noted in a peer-led MAP Canadian model, in which participants reported fewer injuries, overdoses, and hospitalizations related to a decreased consumption of NBA and other drugs compared to prior to MAP participation [29].

An increase in feelings of personal safety and security by Canadian MAP participants was noted throughout four studies [36, 50, 53, 54]. The authors of these studies found MAP participants reported increased feelings of safety and security as compared to other places including streets, shelters, jails, and hospitals. In one study of a day-only non-residential program, researchers found that participants and staff attending the day program expressed concern regarding aggression by fellow participants. Yet participants and staff often preferred to de-escalate behavioral difficulties without involving law enforcement; participants stated aggression occurring within the MAP was less harmful than those in the community [46].

Importantly, in a longer-term perspective, participants expressed an increase in both feelings of community within the MAP and positive social relationships [29, 36, 46, 49, 50, 54, 55]. These results suggest the ability to rebuild a community and social network of friends was mediated by staff acceptance and support and a perceived commonality with other residents. Access to very low-barrier employment opportunities within the MAP community likewise positively impacted individual’s relations and provided transferable skills including leadership training [46].

Lastly, participants and staff of MAPs spoke of the importance of reconciling the external and societal stigma faced as a person with a severe AUD. In three studies, Canadian participants reported a reduction in feelings of shame and guilt, largely attributed to the non-stigmatizing and accepting staff and environment [36, 50, 54].

### Feasibility and fit: community level

We included 14 studies that evaluated feasibility and acceptability of MAP at the health systems level [10, 27, 30, 36, 40–44, 47, 53, 56–58]. For communities with an established MAP, qualitative analyses discussed the importance of establishing shared goals and measures of success within and between the MAP and community-based organizations, including holistic measures of health, culture, socioeconomic, social, and priorities defined by participants. Throughout the literature, a number of potential stakeholders were identified as important to involve in the planning and development of a MAP including community-based homeless service providers, medical and behavioral health professionals, community members, law enforcement, culturally focused groups appropriate to the anticipated populations such as Indigenous elders, and potential beneficiaries [10, 27, 42, 43, 47, 58]. Additional efforts may be warranted to find the most appropriate location for a MAP, such as co-location with respite or recuperative care, shelters for persons experiencing homelessness, or transitional housing [27, 40]. Learned best practices included involving participants in developing program policies and allowing alcohol protocols to have some flexibility and be tailored to the individual [10, 27, 30, 36, 43, 47, 53, 56]. In an overview of site selection and facility layout, importance was placed on ensuring accessibility to public transportation, providing outdoor space, a large dining area, and rooms available for meetings [10, 40, 59].

Researchers of the remaining studies focused on regions yet to establish MAPs. Using qualitative methods to survey key stakeholders (e.g., potential MAP participants and social services personnel), they found high acceptability of establishing a MAP in Sydney Australia, Scotland UK, and London Canada [40–43]. Researchers based potential models off the Canadian MAPs. Stakeholders favored the increased safety, security, and privacy that MAPs could potentially offer and the role in improving supportive relationships including increasing access for medical care. Researchers of a mixed-methods study in Scotland UK noted novel findings compared to Sydney, Australia, and London, Canada. Per interviews, participants found a preference for a drop-in MAP model over residential as it was seen as low threshold, more flexible, less restrictive, and could accommodate a smoother transition from street into residential settings. Perceived benefits of residential settings were the ability for around-the-clock care and a direct resolution of homelessness by providing transitional or permanent housing [40]. Lastly, there was disagreement if a MAP would be the final permanent housing for an individual versus operating as a transitional model with an expectation for discharge to and re-integration to standard housing as an exit [42, 43].

However, researchers of one qualitative study of addiction counselors from Poland found low acceptability and belief that a MAP would not be feasible at that time [44]. Researchers found that acceptance of harm reduction principles around alcohol use was lacking and noted that cultural and interpersonal stigmas of addiction needed to be addressed before MAP establishment. Several research groups also looked at MAP acceptability among Indigenous communities in Canada, who share a disproportionate burden of AUD. Indigenous populations face centuries of oppression from colonialism and racism, resulting in cumulation of both individual and generational trauma. Alcohol use can help individuals cope with such trauma in the short-term but may lead to “social dislocation” and individual isolation among individuals who develop AUD [60]. Interviews with stakeholders from Indigenous communities also found MAPs to be highly acceptable and emphasized the need for program frameworks to reconnect individuals into their social and familial networks in culturally informed ways that engaged the community in all stages of recovery and healing [57, 58].

### Key recommendations of the literature for MAP implementation

Collectively, the literature offered several key recommendations in the design, development, and operation of MAPs.

#### Population selection

Though the literature lacked specific details on how potentially eligible participants are referred into MAP services, criteria aimed at population selection were outlined throughout the work. As noted by Pauly et al. [10] in an overview of Canadian MAPs, common eligibility requirements include: a history of hazardous drinking (e.g., binge drinking or consumption of NBA), multiple attempts at treatment, homelessness, and/or a high use of emergency department services and/or numerous contacts with law enforcement. Assessment via validated tools may increase accuracy, with the Alcohol Use Disorders Identification Test (AUDIT) administered in 11 of 13 programs [10]. Clients selected for a MAP should be at minimum of legal drinking age or older. Additional age or population specifics may be warranted based on the local population, such as accepting clients over a certain age, or prioritizing those who belong to specific racial/ethnic communities, such as Indigenous communities, depending on the stated goals of the MAP and the communities they wish to serve [10]. Considering the importance of prioritizing those who may most benefit by MAP services, in relation to other vulnerable individuals in the community, the referral process was largely not specified in the literature. Additional details on the admission process and how agencies identify and refer potential participants may be helpful.

#### Facility and operations

Flexibility of both layout and design of a MAP was highlighted in studies, though a few specific recommendations were noted. First, create a separation between existing, stabilized MAP clients and newer, incoming residents. This may involve distinct sleeping areas, common spaces, and/or for alcohol distribution [56]. Second, recommendations from a comprehensive review of Canadian MAPs included a straightforward floorplan offering access, ability for observation, common and dining spaces, and access to the outdoors [10, 61]. Programs should plan for the additional onsite space and staffing required for the storage and distribution of alcohol to the residents. Yet this evaluation of Canadian MAPs likewise indicates a need to improve services for all genders by both incorporating women into the planning of these services and applying a “gender lens” to program development [10]. Additional work identified a need for space and programming inclusive of nonbinary gender and LGBTQ + persons [46].

In addition to maintaining consistent communication between all stakeholders, Canadian MAP staff and local community stakeholders highlighted the importance of continuous training on the needs of served individuals with severe AUD. They recommended focusing education on alcohol harm reduction and goals of the MAP for both MAP staff and the wider community. Additionally, they emphasize the importance of offering opportunities for ongoing communication and feedback between staff and MAP residents. This can be in the form of regular within-MAP community meetings between residents and staff, collaboration between staff and residents in day-to-day tasks, and by offering peer leadership opportunities for residents to participate in programmatic and operational decision-making [36, 46, 56].

#### Involving peer/residents and community members in MAP programming

Involving the expertise and perspective of peers was cited frequently by both program leadership and participants as a critical priority, including creating and sustaining peer leadership roles and educating other staff to the value of peer leaders offering their expertise [10, 29, 46]. One notable challenge was identified for peer staffing. In a community peer-based Canadian MAP, many community members working as peers were not able to separate work and home; they found their roles seemed to extend 24 h a day, 7 days a week [46]. Though the researchers did not directly address solutions to this challenge, future efforts could implement methods to discuss and balance the role of peer-level staff both within and outside their professional responsibilities. Additionally, ensuring that advisory groups to MAP programs also include members from the surrounding neighborhoods and communities can improve the likelihood that programs are accepted within their larger surroundings and that community concerns are addressed—both of which may improve program sustainability [62].

Specific to Indigenous populations, three primary recommendations aimed to successfully reconnect MAP participants to their cultural roots. The insight and involvement of local Indigenous leadership is critical to developing a care approach that extends both within and outside the MAP into the community. Researchers recommended proactively engaging the local community of Indigenous persons in MAP development and decision-making processes. Second, incorporate elders and peers directly into the care model. This supports knowledge sharing, ongoing peer support, availability of role models within the culture, and access to healing practices found in traditional ceremony. Lastly, in MAPs focused on Indigenous individuals, leadership and front-line staffing roles should likewise include persons from these communities [10, 57, 58]. A Canadian qualitative review noted variance between peers on whether individuals actively intoxicated may be permitted to participate in Indigenous practices; most did agree flexibility is warranted with all individuals invited to be present even if they were not permitted to participate [46].

#### Managing alcohol consumption

The management of alcohol distribution and consumption is unique to both individual participants and programs. Muckle et al. noted the initial practice of serving a single standard drink every 1 h resulted in high intoxication levels and behavioral challenges [56]. At the program-level, findings suggest stringent policies aimed at non-MAP alcohol consumption are more effective at reducing outside alcohol consumption as opposed to more lax policies [29]. Assessment for over-intoxication prior to alcohol administration is recommended, with current alcohol dosing withheld until the client is less visibly intoxicated. Finally, most Canadian MAPs do allow for clients to purchase their drink-of-choice for dispensing [10].

## Discussion

In our scoping review, we evaluated both the scientific and gray literature to answer the questions, “*What factors are associated with a successful MAP, from the perspective of client outcomes?* And *what are the factors related to MAPs that are perceived as making them a good fit for clients and for communities?”* Though an emerging area of study, the literature offered very promising input and evaluation of the use of MAPs in the stabilization of individuals experiencing homelessness with severe AUD. Our review found that MAPs may decrease alcohol-related harms and improve outcomes among individuals with severe AUD (including early evidence of reduced overall alcohol consumption, improved housing retention, and improved quality of life), while other areas particularly focused on long-term evaluation and implementation require further study.

Considering the devastating effects of severe AUD and co-occurring homelessness, from a research perspective, we need to explore outcomes that are holistic and realistic. We found substantial benefits to well-being as reported by MAP participants, including a notable increase in feelings of safety and security, reconnection with community and a sense of belonging, an increase in self-efficacy, and a positive reconciliation of the internalized shame and stigma related to severe AUD. Individuals with severe AUD and homelessness have higher rates of mortality often resulting in early death. Research into homeless-related deaths find that in 30% of individuals who are unsheltered, mortality is attributable directly to AUD, with up to 50% attributable to combined alcohol and substance use [7, 63].

Yet, caution should be taken when evaluating a causal effect of MAP on participant mortality. As noted, efforts are made to only include individuals with severe AUD where daily, managed consumption of alcohol will not increase harms [10, 45]. Individuals who fit the criteria for a MAP are likely to have preexisting negative health effects from their long-term alcohol consumption; this high risk of mortality may not necessarily be truncated despite MAP participation. Researchers found in a longitudinal cohort study of older adults with substance use disorder who become homeless after the age of 50, their co-occurring chronic medical conditions, trauma history, and lack of healthcare may impact mortality regardless of supports received later in life [64, 65]. Future work may consider a more nuanced look into the process and occurrence of death, including connection to palliative or hospice care, location of death (e.g., on the street, in a hospital, at home in MAP), and successful adherence to end-of-life preferences by the individual. Lastly, the operation of a MAP may directly reduce deaths which occur on the street by individuals who are experiencing homelessness and die outside, unhoused, and without separation from their alcohol use. Yet, despite the longevity of some of the MAPs, we did not find enough evidence on the progression of alcohol-related morbidity and mortality in a MAP. Though a few studies did mention the occurrence of client deaths in MAP during study periods, there were no studies comparing the mortality rate and reasons of death within a MAP as compared to control environments (such as a shelter, respite, or those unsheltered). In a study published after the time parameter of this review, researchers conducted a large retrospective cohort study evaluating mortality and healthcare utilization for MAP participants. They evaluated both within-subject for times the participants were either within and outside of MAP and then a comparison of MAP participants to community controls. Offering promising evidence, they found that participation in MAP did not increase mortality as compared to controls [66]. When they compared within-subject, MAP participants had a significantly lower mortality risk while in versus outside of MAP. Additionally, MAP participation may offer a level of health protection as evidenced by significant fewer hospitalizations as compared to controls [66].

Harm reduction is a critical aspect of managed alcohol, stated throughout most of the featured literature. As seen through this review, participation in a MAP as a harm reduction approach targeting individual risk reduction may directly affect the consumption of alcohol, in particular stabilizing use and reducing binge drinking. Despite the findings on changes in alcohol consumption, there was limited information regarding the management of drug use within a MAP or consumption changes as compared to use prior to MAP participation, presenting an area for future research. Additionally, MAPs offer a harm reduction-based approach to addressing and resolving structural disparities experienced by most MAP participants. This includes socioeconomic inequities and poverty, housing instability and homelessness, victimization, trauma, and intense societal stigma placing culpability of circumstance on the individual. By creating community within a MAP, and providing trauma informed care, the MAP offers both a safe space for participants to rebuild the self and the support to reduce disparities and provide re-integration to society.

Housing retention was a notable feature of the literature with most programs offering permanent housing. As found in the Housing First literature, low-barrier access to housing incorporating a harm reduction philosophy has been shown to be effective in maintaining individuals in housing who have histories of behavioral health diagnoses [67–73]. Yet not all individuals sustain housing in Housing First, and despite the widely accepted success of Housing First, these exits from housing are understudied [74–76]. Separate from residential programs, there is a lack of sufficient evidence for the role of MAPs outside of project-based permanent housing. Additional research is needed to evaluate day-only or scattered-site mobile MAP services. The data so far indicate that, though MAP outside of a residential setting may be feasible, the benefit of accessing day-only MAP services may be diminished by the harms of being unhoused and having nowhere safe to go at night.

Our review found several potential health- and harm reduction-related benefits of MAPs, including improved quality of life, reduced alcohol consumption particularly with decreased NBA use (particularly for programs with Stringent outside drinking policies compared to Lax), less hazardous consumption patterns, and potential stabilizations in biological markers of alcohol use during MAP participation. Decreased consumption of both beverage alcohol and NBA has several downstream benefits, including fewer traumas, assaults, seizures, hazards from NBA including ethanol, higher alcohol content, and additive ingredients [77], and effects of acute intoxication. Potential harms of continued alcohol consumption that are not yet represented in the MAP literature include the progression of liver disease and cirrhosis, cancer, hypertension, or cardiac disease [1, 7, 78–81]. Alcohol-related outcomes that may benefit from further research include the effects on survival behaviors (e.g., need to panhandle, thefts), and negative outcomes from intoxication (e.g., rates of falls, seizures, traumatic brain injury). One approach to assess these outcomes is to ensure collection of a minimum and ideally standardized set of data points by communities developing and operating MAPs. These data could include healthcare diagnoses and utilization patterns prior to and during MAP participation; incoming survey of self-reported consumption, survival behaviors, and alcohol-related harms; or community contacts with law enforcement, shelter access, or drop-in centers. Though it is likely not feasible for program staff to provide evaluation, standardized data collection and availability offers comparative evaluation capability. Considering the impact outside drinking policy differentials (Lax vs. Stringent) appear to have on consumption, future work may include the development and inclusion of a standardized definition ranking drinking policies on a stringency continuum. Researchers could utilize this information in the background of the program, offering a level of comparison with future studies, which will lead to additional findings indicating the potential influences on consumption. The research featured thus far offers substantial support to MAPs in their positive role in improving health and harm reduction outcomes.

Stigma associated with alcohol and other drug use disorders was a theme throughout, both from the participant perspective and in relation to the development and operation of a MAP within the community. Stigma associated with AUD remains a challenge both externally toward the individual with AUD and internalized self-stigmatizing beliefs [82–85]. Our findings indicate that MAPs may successfully decrease this internalized stigma through specific actions, including offering peer-level and appropriately trained staff, offering low-barrier, non-judgmental environments, and building community among MAP participants. This engagement is critical not only for developing patient-centered models tailored to the cultural needs of participants, but ongoing education and collaboration could also support relations between community and program. However, as noted in the Poland study, this persistent and lingering reality may negatively impact the ability to achieve provider or community buy-in to consider a MAP. Thus, substantial upstream work within the community, including within the healthcare system, may be required prior to developing and implementing MAPs, and future studies should elucidate challenges and best practices for community engagement.

### Limitations

We made considerable efforts to provide a comprehensive review of MAPs, yet weaknesses may impact our findings. Though all attempts were made to correctly compare findings between studies and gray literature, errors may have been made. To reduce this potential, our team included multiple reviewers and followed PRISMA guidelines that increase our validity. And although no language restrictions were placed for the gray literature search, both search engines—Google and Ecosia—were accessed from a browser set in the English language. This may have skewed results to those in English. Lastly, as a scoping review is intended to provide a broad overview of the body of literature concerning MAPs and identify knowledge gaps, we did not conduct a critical appraisal of the specific findings. Lastly, most studies originate from Canada, potentially limiting the applicability and generalizability to cultures and communities outside of Canada.

A considerable challenge is developing measures of success in which to evaluate the impact of MAPs [86], and there are a few methodological challenges (e.g., reliance on self-reporting, obtaining adequate pre-MAP health data for pre–post evaluations) facing researchers who wish to further explore the impact of MAPs and address gaps in the literature. As the topic of managed alcohol is a growing phenomenon, the methods of evaluation for a MAP are not yet clearly defined or standardized and often relies on self-reported outcomes. Self-reported data are not inherently negative, yet—as was noted in the research discussing non-MAP, outside consumption of alcohol—there can be stark differences in self-reporting based on individual program operations. A second area relates to the method of population selection and determining who is the best fit for a MAP. There was limited work discussing or evaluating the rigor of the MAP referral models in practice, which will offer important insights to communities creating their own service models. Lastly, at the time of this review, there are limited longitudinal data to evaluate longer-term implications of MAP participation. A potential challenge has been the ability to harness comprehensive data of individuals before their MAP participation, at MAP admission, and throughout their time within a MAP or post-MAP for those who depart. Longitudinal follow-up can explore physical and mental health-related measures, cross-system linkages to identify service patterns, and ensure appropriate comparisons are made to control groups. This information will help answer lingering questions of whether MAP participation itself increases specific harms or introduces new alcohol-related harms.

## Conclusion

Our objectives were to characterize key components of MAPs, identify the intended and measured impacts of a MAP, identify gaps in knowledge and the literature, and recommend possible areas for future review. The growing literature showcases several outcomes of interest, with increasing efforts aimed at identifying the most appropriate measures by which to determine the effectiveness and potential risks of MAP participation. Based in a harm reduction approach, MAPs offer a promising, targeted intervention for individuals suffering from severe AUD and co-occurring homelessness.

## Data Availability

Not applicable.

## Appendix 1: Preferred reporting items for systematic reviews and meta-analyses extension for scoping reviews (PRISMA-ScR) checklist

**Table:**

SECTION	ITEM	PRISMA-ScR CHECKLIST ITEM	REPORTED ON PAGE #
TITLE			
Title	1	Identify the report as a scoping review	1
ABSTRACT			
Structured summary	2	Provide a structured summary that includes (as applicable): background, objectives, eligibility criteria, sources of evidence, charting methods, results, and conclusions that relate to the review questions and objectives	1–2
INTRODUCTION			
Rationale	3	Describe the rationale for the review in the context of what is already known. Explain why the review questions/objectives lend themselves to a scoping review approach	1
Objectives	4	Provide an explicit statement of the questions and objectives being addressed with reference to their key elements (e.g., population or participants, concepts, and context) or other relevant key elements used to conceptualize the review questions and/or objectives	1
METHODS			
Protocol and registration	5	Indicate whether a review protocol exists; state if and where it can be accessed (e.g., a Web address); and if available, provide registration information, including the registration number	n/a
Eligibility criteria	6	Specify characteristics of the sources of evidence used as eligibility criteria (e.g., years considered, language, and publication status), and provide a rationale	Page 2
Information sources*	7	Describe all information sources in the search (e.g., databases with dates of coverage and contact with authors to identify additional sources), as well as the date the most recent search was executed	Page 1, Appendix 3
Search	8	Present the full electronic search strategy for at least 1 database, including any limits used, such that it could be repeated	Appendix 3
Selection of sources of evidence^†^	9	State the process for selecting sources of evidence (e.g., screening and eligibility) included in the scoping review	1,2
Data charting process^‡^	10	Describe the methods of charting data from the included sources of evidence (e.g., calibrated forms or forms that have been tested by the team before their use, and whether data charting was done independently or in duplicate) and any processes for obtaining and confirming data from investigators	2
Data items	11	List and define all variables for which data were sought and any assumptions and simplifications made	2
Critical appraisal of individual sources of evidence^§^	12	If done, provide a rationale for conducting a critical appraisal of included sources of evidence; describe the methods used and how this information was used in any data synthesis (if appropriate)	n/a
Synthesis of results	13	Describe the methods of handling and summarizing the data that were charted	2
RESULTS			
Selection of sources of evidence	14	Give numbers of sources of evidence screened, assessed for eligibility, and included in the review, with reasons for exclusions at each stage, ideally using a flow diagram	Figure 1
Characteristics of sources of evidence	15	For each source of evidence, present characteristics for which data were charted and provide the citations	Tables 1–3
Critical appraisal within sources of evidence	16	If done, present data on critical appraisal of included sources of evidence (see item 12)	n/a
Results of individual sources of evidence	17	For each included source of evidence, present the relevant data that were charted that relate to the review questions and objectives	Tables 1–3
Synthesis of results	18	Summarize and/or present the charting results as they relate to the review questions and objectives	Pages 2–6, Tables 1–3
DISCUSSION			
Summary of evidence	19	Summarize the main results (including an overview of concepts, themes, and types of evidence available), link to the review questions and objectives, and consider the relevance to key groups	6–8
Limitations	20	Discuss the limitations of the scoping review process	8
Conclusions	21	Provide a general interpretation of the results with respect to the review questions and objectives, as well as potential implications and/or next steps	8
FUNDING			
Funding	22	Describe sources of funding for the included sources of evidence, as well as sources of funding for the scoping review. Describe the role of the funders of the scoping review	1

*From:* Tricco AC, Lillie E, Zarin W, O'Brien KK, Colquhoun H, Levac D, et al. PRISMA Extension for Scoping Reviews (PRISMA-ScR): Checklist and Explanation. Ann Intern Med.;169:467–473. https://doi.org/10.7326/M18-0850

*JBI* Joanna Briggs Institute, *PRISMA-ScR* preferred reporting items for systematic reviews and meta-analyses extension for scoping reviews

*Where *sources of evidence* (see second footnote) are compiled from, such as bibliographic databases, social media platforms, and Web sites

^†^A more inclusive/heterogeneous term used to account for the different types of evidence or data sources (e.g., quantitative and/or qualitative research, expert opinion, and policy documents) that may be eligible in a scoping review as opposed to only studies. This is not to be confused with *information sources* (see first footnote)

^‡^The frameworks by Arksey and O’Malley (6) and Levac and colleagues (7) and the JBI guidance (4, 5) refer to the process of data extraction in a scoping review as data charting

^§^The process of systematically examining research evidence to assess its validity, results, and relevance before using it to inform a decision. This term is used for items 12 and 19 instead of "risk of bias" (which is more applicable to systematic reviews of interventions) to include and acknowledge the various sources of evidence that may be used in a scoping review (e.g., quantitative and/or qualitative research, expert opinion, and policy document)

## Appendix 2: Preferred reporting items for systematic reviews and meta-analyses (PRISMA) checklist

**Table:**

Section/topic	#	Checklist item	Location(s) reported
INFORMATION SOURCES AND METHODS			
Database name	1	Name each individual database searched, stating the platform for each	p 1, Appendix 3
Multi-database searching	2	If databases were searched simultaneously on a single platform, state the name of the platform, listing all of the databases searched	n/a
Study registries	3	List any study registries searched	n/a
Online resources and browsing	4	Describe any online or print source purposefully searched or browsed (e.g., tables of contents, print conference proceedings, web sites), and how this was done	n/a
Citation searching	5	Indicate whether cited references or citing references were examined, and describe any methods used for locating cited/citing references (e.g., browsing reference lists, using a citation index, setting up email alerts for references citing included studies)	2
Contacts	6	Indicate whether additional studies or data were sought by contacting authors, experts, manufacturers, or others	2
Other methods	7	Describe any additional information sources or search methods used	2
SEARCH STRATEGIES			
Full search strategies	8	Include the search strategies for each database and information source, copied and pasted exactly as run	Appendix 3
Limits and restrictions	9	Specify that no limits were used, or describe any limits or restrictions applied to a search (e.g., date or time period, language, study design) and provide justification for their use	p 1, Appendix 3
Search filters	10	Indicate whether published search filters were used (as originally designed or modified), and if so, cite the filter(s) used	n/a
Prior work	11	Indicate when search strategies from other literature reviews were adapted or reused for a substantive part or all of the search, citing the previous review(s)	n/a
Updates	12	Report the methods used to update the search(es) (e.g., rerunning searches, email alerts)	p 1, Appendix 3
Dates of searches	13	For each search strategy, provide the date when the last search occurred	p 1, Appendix 3
PEER REVIEW			
Peer review	14	Describe any search peer review process	n/a
MANAGING RECORDS			
Total Records	15	Document the total number of records identified from each database and other information sources	Appendix 3, Fig. 1
Deduplication	16	Describe the processes and any software used to deduplicate records from multiple database searches and other information sources	Appendix 3
PRISMA-S: an extension to the PRISMA statement for reporting literature searches in systematic reviews			
Rethlefsen ML, Kirtley S, Waffenschmidt S, Ayala AP, Moher D, Page MJ, Koffel JB, PRISMA-S Group			
Last updated February 27, 2020			

## Appendix 3: All searches conducted on November 14, 2019 and updated on April 1, 2021. No language or date limits used. De-duplication was completed in EndNote X9 on November 14, 2019

**Table:**

Database	Search strategy	Number of results
PubMed (1966-)	"managed alcohol"[tiab] OR "surrogate alcohol"[tiab]	57
Embase (1947-)	"managed alcohol" OR "wet shelter" OR "wet shelters" OR ('controlled alcohol' AND program*) OR "surrogate alcohol"	79
CINAHL Complete (EBSCO, 1937-)	"managed alcohol" OR "surrogate alcohol" OR "controlled alcohol"	56
PsycINFO (ProQuest, 1887-)	"managed alcohol" OR "wet housing" OR "wet shelter" OR "wet shelters" OR "controlled alcohol"	65
Sociological Abstracts & Social Services Abstracts (searched together via ProQuest: 1963-)	"managed alcohol" OR "wet housing" OR "wet shelter" OR "wet shelters" OR "controlled alcohol"	15
Google Scholar	"wet housing" OR "wet shelter" OR "wet shelters" OR "managed alcohol" OR ("alcohol management" AND housing) OR "controlled alcohol" OR "surrogate alcohol"	150
Total number of results		422
Total number of duplicates		143
Total number after de-duplication		279

## Abbreviations

AUD: Alcohol use disorder
COVID-19: Coronavirus disease 2019
MAP: Managed alcohol program
NBA: Non-beverage alcohol
PRISMA-ScR: Preferred reporting items for systematic reviews and meta-analyses guidelines—extension for scoping reviews
WHO: World Health Organization
